# A Truncated Receptor-Binding Domain of MERS-CoV Spike Protein Potently Inhibits MERS-CoV Infection and Induces Strong Neutralizing Antibody Responses: Implication for Developing Therapeutics and Vaccines

**DOI:** 10.1371/journal.pone.0081587

**Published:** 2013-12-04

**Authors:** Lanying Du, Zhihua Kou, Cuiqing Ma, Xinrong Tao, Lili Wang, Guangyu Zhao, Yaoqing Chen, Fei Yu, Chien-Te K. Tseng, Yusen Zhou, Shibo Jiang

**Affiliations:** 1 Lindsley F. Kimball Research Institute, New York Blood Center, New York, New York, United States of America; 2 State Key Laboratory of Pathogen and Biosecurity, Beijing Institute of Microbiology and Epidemiology, Beijing, China; 3 Departments of Microbiology and Immunology, University of Texas Medical Branch, Galveston, Texas, United States of America; 4 Department of Pharmacology, University of Minnesota Medical School, Minneapolis, Minnesota, United States of America; 5 Key Laboratory of Medical Molecular Virology of Ministries of Education and Health, Shanghai Medical College and Institute of Medical Microbi, Fudan University, Shanghai, China; The University of Hong Kong, Hong Kong

## Abstract

An emerging respiratory infectious disease with high mortality, Middle East respiratory syndrome (MERS), is caused by a novel coronavirus (MERS-CoV). It was first reported in 2012 in Saudi Arabia and has now spread to eight countries. Development of effective therapeutics and vaccines is crucial to save lives and halt the spread of MERS-CoV. Here, we show that a recombinant protein containing a 212-amino acid fragment (residues 377-588) in the truncated receptor-binding domain (RBD: residues 367–606) of MERS-CoV spike (S) protein fused with human IgG Fc fragment (S377-588-Fc) is highly expressed in the culture supernatant of transfected 293T cells. The purified S377-588-Fc protein efficiently binds to dipeptidyl peptidase 4 (DPP4), the receptor of MERS-CoV, and potently inhibited MERS-CoV infection, suggesting its potential to be further developed as a therapeutic modality for treating MERS-CoV infection and saving the patients’ lives. The recombinant S377-588-Fc is able to induce in the vaccinated mice strong MERS-CoV S-specific antibodies, which blocks the binding of RBD to DPP4 receptor and effectively neutralizes MERS-CoV infection. These findings indicate that this truncated RBD protein shows promise for further development as an effective and safe vaccine for the prevention of MERS-CoV infection.

## Introduction

A novel human coronavirus, Middle East respiratory syndrome (MERS) coronavirus (MERS-CoV), was identified as a pathogen causing a severe acute respiratory syndrome (SARS)-like disease in the Middle East and Europe in 2012 [1]. As of October 14, 2013, the World Health Organization (WHO) had been informed of 138 confirmed cases of MERS-CoV infection, including 60 deaths (a case fatality rate of 45%) (http://www.who.int/csr/don/2013_10_14/en/). Recent reports of family clusters and health care-associated transmission of MERS-CoV through close contact have proven its capacity for human-to-human transmission [2]–[5]. Although its transmissibility is significantly lower than that of SARS coronavirus (SARS-CoV) [6]–[9], it may gain increased human-to-human transmissibility during its further evolution and potentially cause a pandemic in the future [10]. Accordingly, development of effective therapeutics and vaccines is critical for early intervention and prevention.

**Figure 1:**
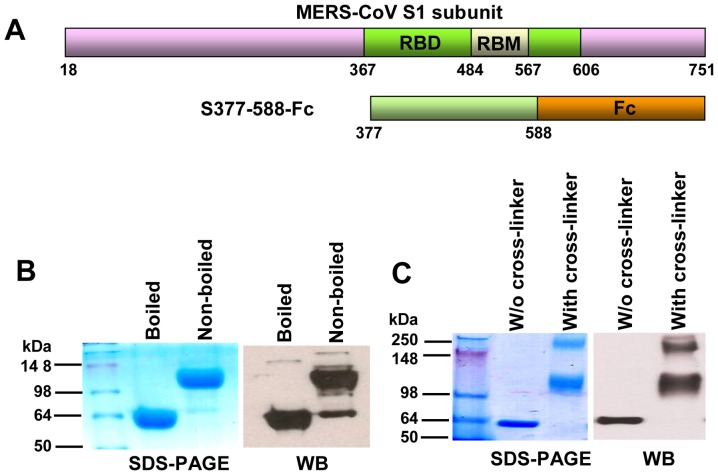
Construction and characterization of MERS-CoV S377-588-Fc. (A) Schematic structure of MERS-CoV S1 subunit and S377-588-Fc. RBM: the receptor-binding motif in the RBD. S377-588-Fc was constructed by fusing MERS-CoV residues 377-588 of S1 with Fc of human IgG. (B) SDS-PAGE and Western blot (WB) analysis of purified 377-588-Fc protein. Samples were either boiled for 10 min, or not boiled, followed by SDS-PAGE (left) and WB (right) analysis using a S1-specific polyclonal antibody. (C) Analysis of S377-588-Fc protein conformation by cross-linker. Samples were cross-linked with glutaraldehyde (with cross-linker at the final concentration of 4 µM) or without cross-linker (w/o cross-linker), followed by SDS-PAGE (left) and WB (right) analysis as described above. The protein molecular weight marker (kDa) (Invitrogen) is indicated on the left.

Unlike SARS-CoV, which uses human angiotensin-converting enzyme 2 (ACE2) as its receptor for binding to ACE2-expressing cells [11], MERS-CoV utilizes a different receptor, dipeptidyl peptidase 4 (DPP4), for binding to DPP4-expressing cells [12]. Like the spike (S) protein of SARS-CoV, the S protein of MERS-CoV also plays important roles in virus entry and infection [13]. MERS-CoV S protein contains a S1 subunit that mediates virus binding to cells expressing DPP4 through its receptor-binding domain (RBD) region and an S2 subunit that mediates virus-cell membrane fusion [12], [13]. Based on sequence alignment and homology modeling analysis and functional studies, we and Mou et al. have predicted that the RBD is located in residues 377-662 or 358-588 of the MERS-CoV S1 subunit [14]–[16] (Fig. 1A). Co-crystallographic analyses of the RBD/DPP4 complexes have confirmed that the RBD is attributed to residues 367-606 or 367-588 in MERS-CoV S1 [17]–[19] (Fig. 1A).

Previous studies have shown that the RBD of SARS-CoV S protein can significantly inhibit SARS-CoV infection [20] and is able to induce highly potent neutralizing antibodies protecting against SARS-CoV infection [20]. It is thus expected that the RBD of MERS-CoV, which belongs to the same betacoronavirus genus as SARS-CoV [21], [22], may also be effective in inhibiting MERS-CoV infection and inducing neutralizing antibody responses against infection of MERS-CoV in vaccinated animals.

Indeed, our identified RBD (a 286-amino acid fragment spanning residues 377-662) could bind to DPP4 and induce neutralizing antibody response in immunized mice [15], while the RBD reported by Mou et al. (a 231-amino acid fragment spanning residues 358-588) could inhibit MERS-CoV infection at the 40 µg/ml level and elicit effective neutralizing antibodies in vaccinated rabbits [16]. These results suggest that the overlapping region (residues 377-588) must contain the receptor-binding motif (RBM) and the major neutralizing epitope of the RBD. Crystallographic analyses indicated that the RBM is located in the middle (residues 484-567) of the RBD [18], [19]. Therefore, we designed a 212-amino acid truncated RBD sequence (residues 377-588) (Fig. 1A) and tested its ability to inhibit MERS-CoV infection and induce neutralizing antibody responses in vaccinated mice in order to identify a relatively optimized RBD sequence for developing anti-MERS-CoV therapeutics and vaccines.

## Results

### Characterization of recombinant protein containing residues 377-588 of MERS-CoV S protein fused with human IgG Fc

We and Mou et al. have shown that the recombinant protein containing residues 377-662 or 358-588 of MERS-CoV S1 and human IgG Fc fragment can induce neutralizing antibody responses in immunized mice or rabbits [15], [16]. We thus believe that a truncated RBD sequence spanning residues 377-588, which includes RBM (residues 484-567) [18], [19], may contain the major neutralizing epitope in RBD. Therefore, we expressed a recombinant protein containing residues 377-588 fused with human IgG-Fc (S377-588-Fc) (Fig. 1A). We found that this protein could be highly expressed in the culture supernatant of transfected 293T cells and purified to high purity. Its molecular weight under the non-boiled condition was almost 1-fold greater than that under boiled condition in 10% SDS-PAGE in the presence of reducing agent 2-mercaptoethanol (Fig. 1B), suggesting that S377-588-Fc formed a stable, dimeric structure under reducing condition.

Cross-linker analysis was then applied to characterize the conformational structure of expressed S377-588-Fc. We found that the molecular weights of the cross-linked S377-588-Fc samples were almost 1- and 3-fold higher than the samples without crosslinking (Fig. 1C), which indicates that MERS-CoV S377-588 fused with Fc tag was able to form dimeric or tetrameric conformational structures. The bands of S377-588-Fc fusion protein in SDS-PAGE were further confirmed by Western blot using a polyclonal antibody against S1 of MERS-CoV (Fig. 1B-C).

### S377-588-Fc protein bound efficiently to DPP4 receptor

**Figure 2:**
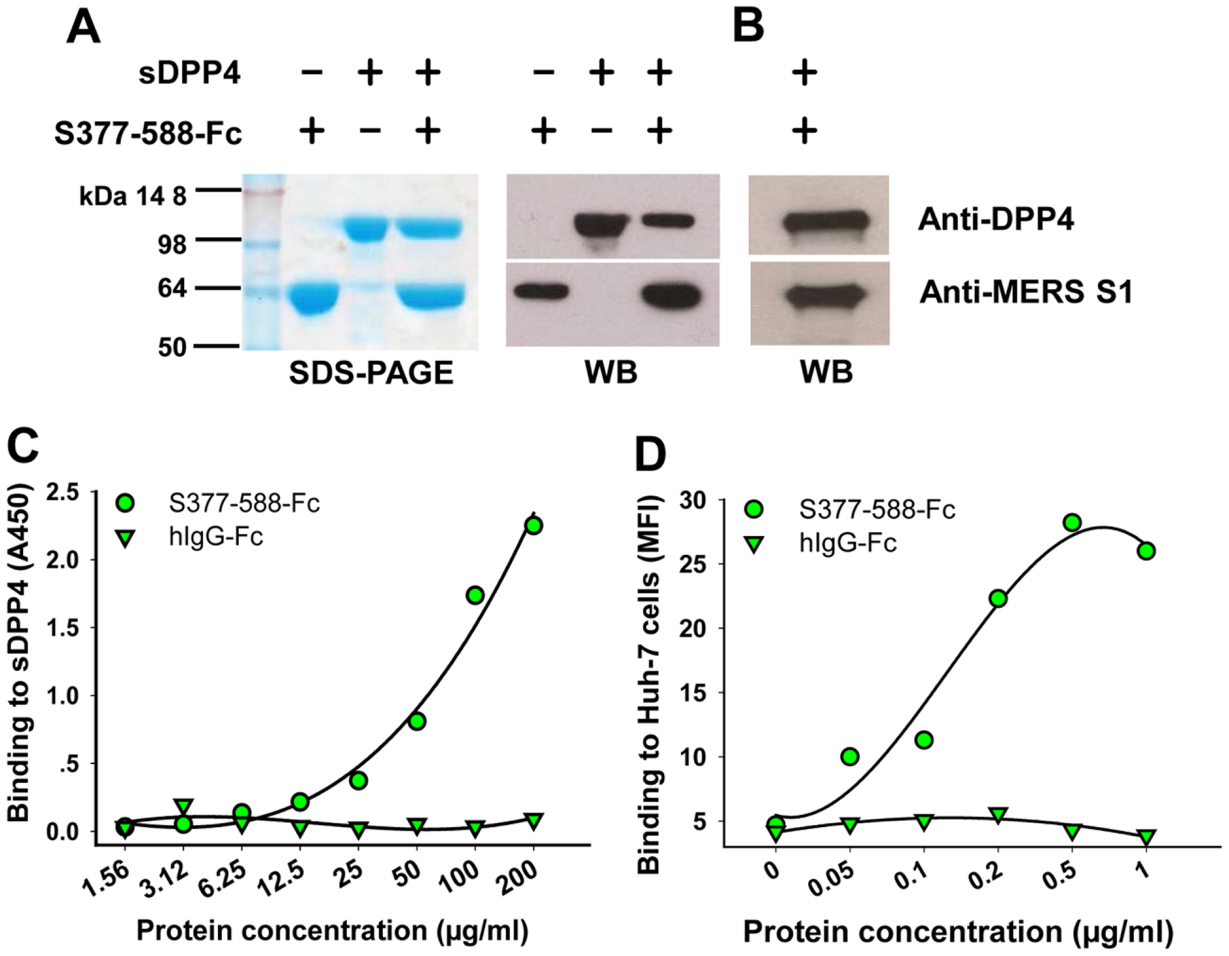
Detection of S377-588-Fc binding to DPP4. Co-immunoprecipitation analysis of S377-588-Fc binding to sDPP4 (A) or DPP4-expressing Huh-7 cells (B). S377-588-Fc protein was mixed with equal concentration of sDPP4 protein (10 µg) or Huh-7 cell lysates in the presence of Protein A Sepharose Beads, followed by detection of the binding by SDS-PAGE and WB using DPP4- (1 µg/ml ) or MERS-CoV S1-specific antibodies (1∶1,000), respectively. The sDPP4 and S377-588-Fc alone were included as the controls. The protein molecular weight marker (kDa) is indicated on the left. Detection of dose-dependent binding of S377-588-Fc to sDPP4 by ELISA (C) or to DPP4-expressing Huh-7 cells by flow cytometry (D). Fc of human IgG (hIgG-Fc) without fusion with MERS-CoV S protein was included as the control. MFI, median fluorescence intensity.

To detect the interaction between S377-588-Fc protein and DPP4, the receptor for MERS-CoV, we first performed a co-immunoprecipitation analysis by mixing equal concentrations of purified S377-588-Fc with soluble DPP4 (sDPP4) in the presence of Protein A Sepharose beads. Next, we analyzed the bound proteins by SDS-PAGE and Western blot using antibodies specific for DPP4 and MERS-CoV S1 proteins, respectively. In SDS-PAGE (Fig. 2A, left), two clear bands respectively corresponding to the size of sDPP4 and S377-588-Fc monomer were shown in the samples co-immunoprecipitated with these two proteins, while only one band corresponding to the size of sDPP4 or S377-588-Fc was indicated in the control sample containing only sDPP4 or S377-588-Fc, respectively. The bands were confirmed by antibodies against DPP4 or MERS-CoV S1, as shown in Western blot (Fig. 2A, right), suggesting the significant binding of S377-588-Fc with sDPP4.

We next detected the interaction between S377-588-Fc protein and cellular DPP4 receptor on DPP4-expressing Huh-7 cells using approaches similar to those described above, but replacing sDPP4 with Huh-7 cell lysates, as we previously described [15]. As expected, two clear bands (corresponding to the size of DPP4 and S377-588-Fc, respectively) were detected from the Protein A beads pre-incubated with the mixture of S377-588-Fc and Huh-7 cell lysates, which reacted strongly with a polyclonal antibody against MERS-CoV S1 and a monoclonal antibody (mAb) specific to DPP4 (Fig. 2B). These results indicated that S377-588-Fc of MERS-CoV was able to bind efficiently to the cell-surface receptor DPP4.

Subsequently, we further detected the binding of S377-588-Fc to sDPP4 by ELISA and cell-associated DPP4 by flow cytometry. As shown in Figure 2C, S377-588-Fc bound sDPP4 that was coated on the ELISA plate in a dose-dependent manner. The flow cytometric analysis also indicated a dose-dependent binding of this protein with DPP4 on DPP4-expressing Huh-7 cells (Fig. 2D). However, the control protein hIgG-Fc did not show significant binding to sDPP4 or cell-associated DPP4 (Fig. 2C-D). These results confirmed the binding specificity of S377-588-Fc to DPP4.

### S377-588-Fc protein effectively inhibited MERS-CoV infection in DPP4-expressing cells

**Figure 3:**
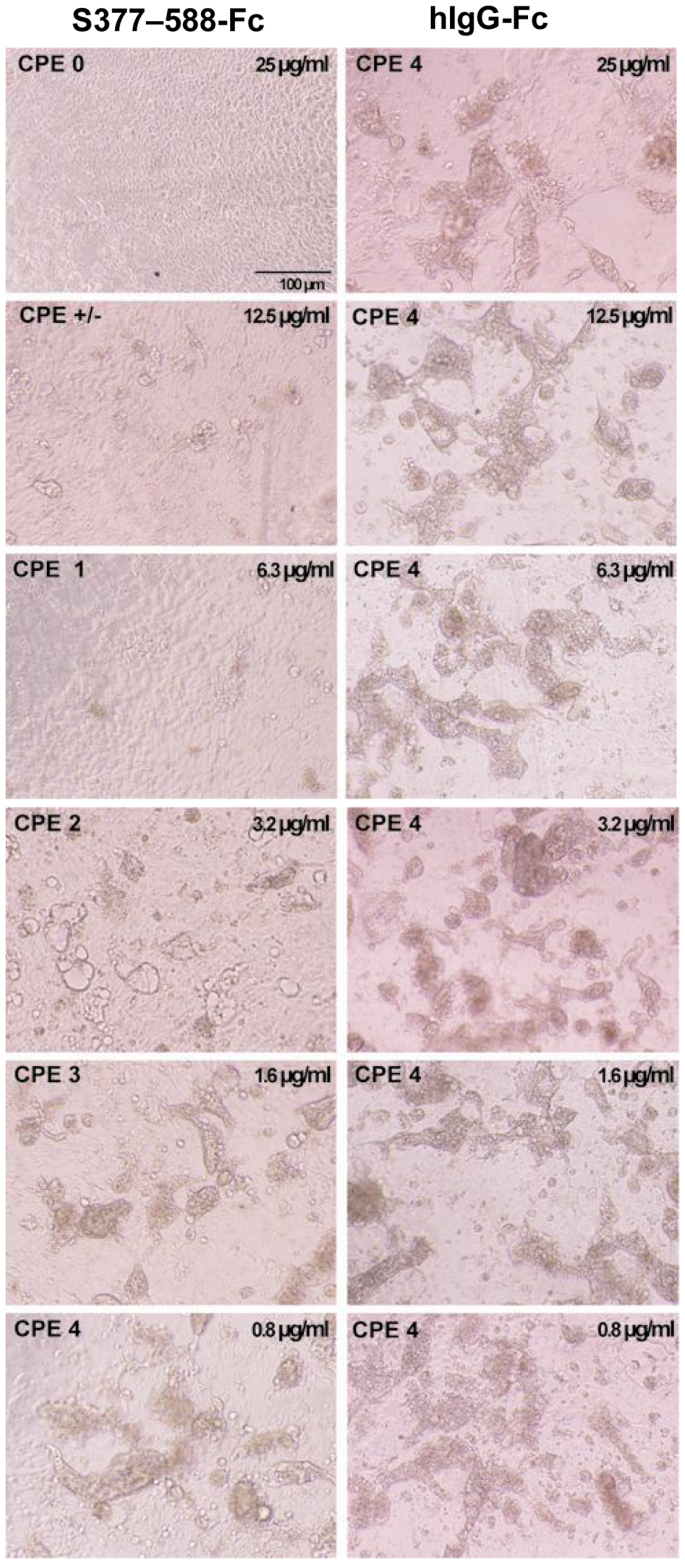
S377-588-Fc protein-mediated inhibition of MERS-CoV infection in Calu-3 cells. hIgG-Fc was included as the control. Prior to addition of MERS-CoV, proteins were incubated with Calu-3 cells. The extent of CPE, ranging from 0 (none), ±(< 5%), 1 (5–10%), 2 (10–25%), 3 (25–50%), and 4 (> 50%), was assessed at 24 h post-infection.

To evaluate the potential inhibitory activity of S377-588-Fc protein against MERS-CoV infection, the highly permissive human bronchial epithelial Calu-3 cells that express DPP4 [23] were incubated with MERS-CoV/EMC-2012 at 0.1 multiplicity of infection (MOI) in the presence of serially diluted S377-588-Fc protein. The viral replication, as indicated by cytopathic effect (CPE) formation, was determined at 24 h post-infection. As shown in Figure 3, S377-588-Fc significantly inhibited MERS-CoV infection in Calu-3 cells in a dose-dependent manner, as indicated by diminished or reduced CPE formation. Specifically, S377-588-Fc at the concentrations of 25 and 12.5 µg/ml could completely, or nearly completely, block CPE formation, respectively, while this protein at 3.2 µg/ml could maintain ≥ 50% inhibition of CPE formation. However, the control hIgG-Fc protein at 25 µg/ml, or even higher (data not shown), exhibited no inhibition of CPE formation. These results suggest that recombinant S377-588-Fc protein has the potential to be further developed as a therapeutic against MERS-CoV infection.

### S377-588-Fc protein induced strong MERS-CoV S-specific antibody responses with neutralizing activities in vaccinated mice

**Figure 4:**
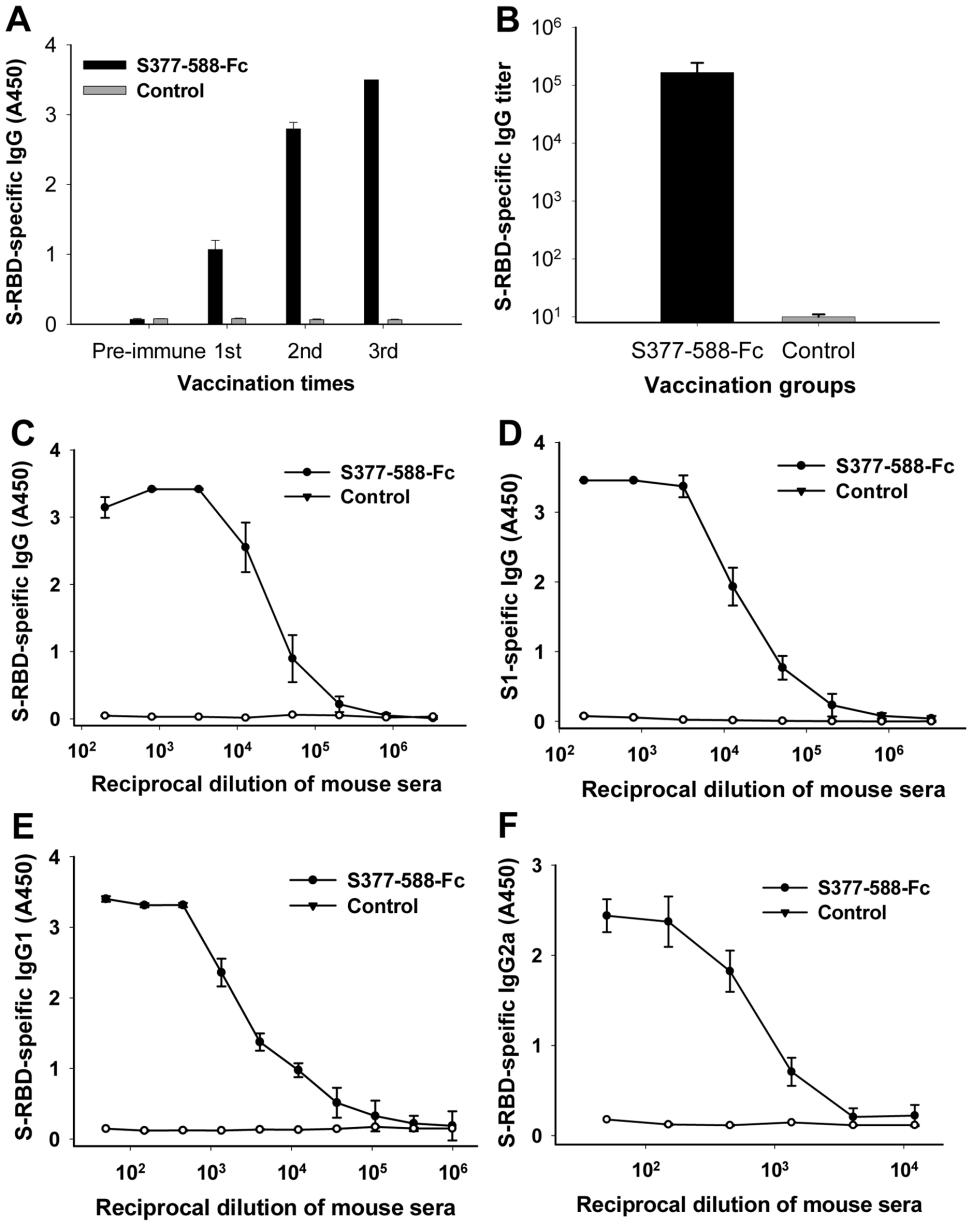
Detection of S-RBD- and S1-specific IgG level in sera of mice immunized with S377-588-Fc protein. PBS was used as the control. (A) The titers of IgG specific to MERS-CoV S377-588-Fc (S-RBD) in sera (1:3,200 dilution) of mice at pre-immunization (pre-immune) and 10 days post-each vaccination. (B) Endpoint IgG titers in mouse sera from 10 days post-last vaccination. The data are presented as mean A450 ± standard deviation (SD) of five mice per group. Ability of IgG binding to MERS-CoV S-RBD (C) and S1 protein (D) was detected using mouse sera from 10 days post-last vaccination. Ability of IgG1 (E) and IgG2a (F) antibodies binding to MERS-CoV S-RBD was detected using sera from 10 days post-last vaccination. The data are presented as mean A450 ± SD of five mice per group at various dilution points.

To evaluate the ability of S377-588-Fc protein to elicit MERS-CoV S-specific antibody responses, we immunized mice subcutaneously (s.c.) using the purified protein in the presence of Montanide ISA 51 adjuvant and detected IgG antibody response, subtypes and neutralizing antibodies in the mouse sera collected at different time points after immunization. The results demonstrated that S377-588-Fc induced increasing IgG antibody responses after each boost, reaching the highest level at day 10 post-3^rd^ vaccination, with the endpoint titer of IgG approaching 1:1.7×10^5^±7.7×10^4^ (Fig. 4A-B). The antibodies in the mouse sera could bind efficiently to both S-RBD fusion protein S377-588-Fc (Fig. 4C) and MERS-CoV S1 protein without Fc (Fig. 4D), suggesting that the antibodies are specific for the RBD in the S1 subunit of MERS-CoV S protein. Detection of IgG subtype antibody responses revealed that S377-588-Fc was able to induce both IgG1 (Th2) and IgG2a (Th1) antibody responses in the vaccinated mice (Fig. 4E-F). However, the PBS control group could not induce significant antibody responses against MERS-CoV S-RBD and S1 proteins (Fig. 4).

**Figure 5:**
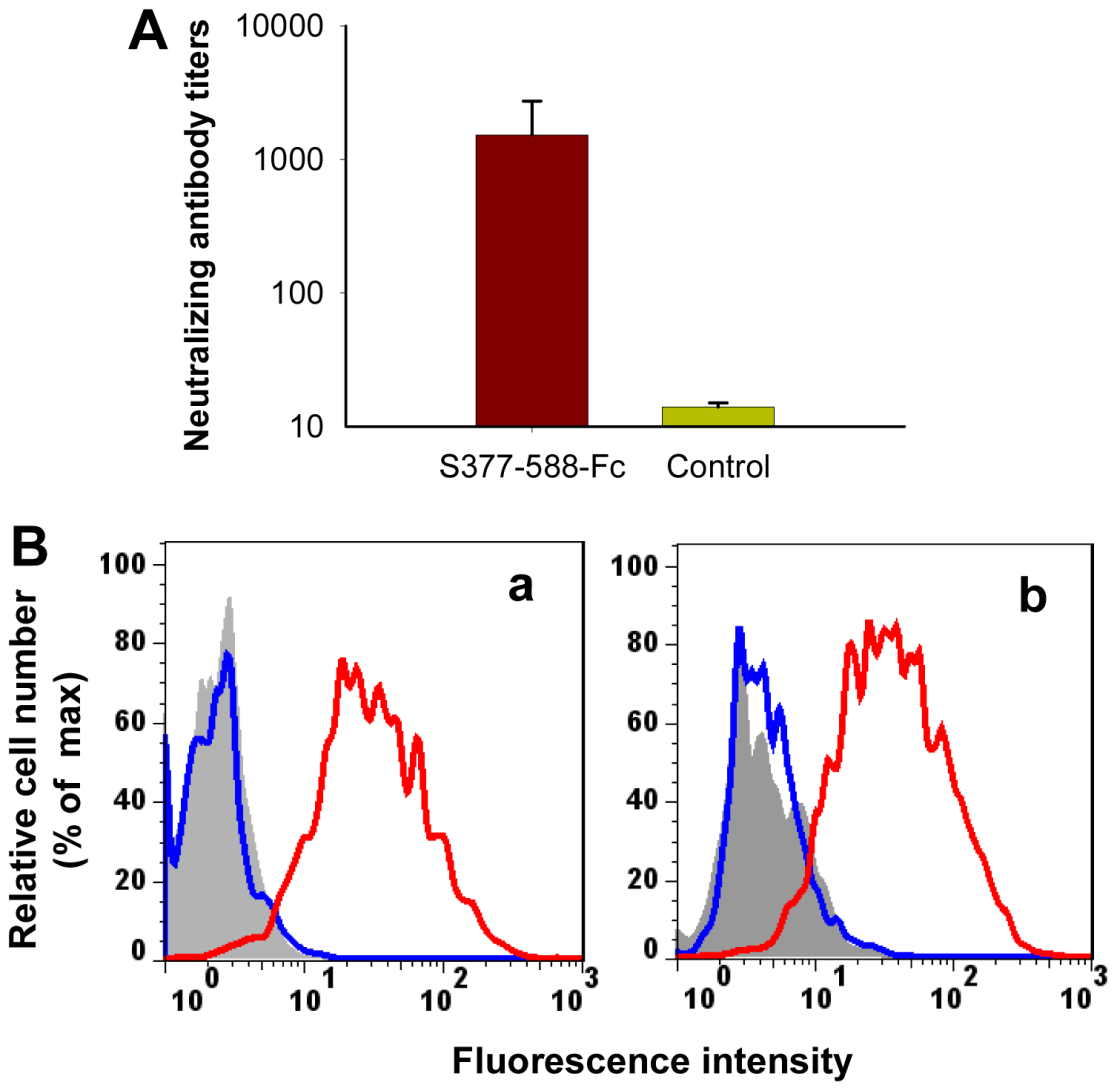
Detection of neutralizing antibodies in S377-588-Fc protein-vaccinated mouse sera and antibody-mediated blockage of receptor binding. (A) Sera collected at 10 days post-last vaccination were tested for neutralization of MERS-CoV infection in Vero E6 cells. PBS was used as the control. Neutralizing antibody titers were expressed as the reciprocal of the highest dilution of serum that completely inhibited virus-induced CPE in at least 50% of the wells (NT_50_), and the results are presented as mean ± SD from five mice per group. (B) Collected mouse sera were tested for blockage of S377-588-Fc binding to DPP4-expressing Huh-7 cells by flow cytometry. (a) The S377-588-Fc protein (red line) bound efficiently to Huh-7 cells (gray shade), while only background binding was shown in the control protein hIgG-Fc (blue line). (b) Blockage of S377-588 binding to Huh-7 cells (gray shade) by vaccinated mouse antisera (1:32, blue line). Sera from the PBS control group (red line) were included as the control. Sera pooled from five mice per group were used for the test.

Neutralizing antibodies in sera of mice at 10 days post-last vaccination with S377-588-Fc protein were evaluated by a live MERS-CoV-based neutralization assay in a Vero E6 cell culture system. As shown in Figure 5A, S377-588-Fc induced high neutralizing antibody responses, with neutralizing antibody titers of 1∶1.5×10^3^±1.2×10^3^, while mouse sera from the PBS control group exhibited no significant neutralizing antibodies against MERS-CoV. These results suggest that S377-588-Fc protein could induce potent anti-MERS-CoV neutralizing antibody responses in the vaccinated animals.

### Antibodies in antisera of mice immunized with S377-588-Fc protein effectively blocked S377-588 binding to DPP4 receptor

To test whether mouse antibodies against S377-588-Fc could block RBD binding DPP4, we incubated S377-588-Fc with DPP4-expressing Huh-7 cells in the presence and absence of mouse antisera, as described in Materials and Methods, followed by flow cytometric analysis. In the absence of antisera, strong signals were detected in S377-588-Fc-incubated Huh-7 cells, while only background signals were observed in the control hIgG-Fc-incubated cells (Fig. 5Ba), confirming that the truncated RBD (S377-588) could specifically bind to the DPP4-expressing cells. However, in the presence of antisera against S377-588-Fc, the binding of S377-588 to DPP4-expressing Huh-7 cells was effectively blocked, while the control sera from PBS-vaccinated mice showed no significant inhibition on the binding of S377-588 to DPP4-expressing Huh-7 cells (Fig. 5Bb).

## Discussion

Because of the high mortality of the SARS-like disease caused by MERS-CoV and its human-to-human transmissibility [4], development of effective therapeutics and vaccines for intervention and prevention is urgently needed [10]. Previous studies have shown that the RBD of SARS-CoV is one of the most important targets for developing SARS-CoV entry inhibitors and SARS vaccines [20], [24]–[27]. Therefore, we used similar approaches to identify the optimal sequence of RBD in MERS-CoV S protein as a target for developing anti-MERS therapeutics and vaccines.

Several groups have independently solved the crystal structure of MERS-CoV S-RBD (residues 367-588 or 367-606), which contains the RBM (residues 484-567) (Fig. 1A) [17]–[19]. Mou et al. and our group have shown that the recombinant proteins containing residues 358-588 and 377-662, respectively, could induce MERS-CoV neutralizing antibody responses in immunized animals [15], [16], suggesting that the overlapping region, which spans residues 377-588, must contain the major neutralizing epitope. Based on these findings, we designed a truncated RBD fragment containing residues 377-588, which contains the RBM (residues 484-567) of MERS-CoV S protein S1 subunit, and expressed a recombinant protein covering this region fused with Fc of human IgG. Fusion of Fc tag with S377-588 was carried out in order to 1) facilitate the affinity-purification of the Fc-containing fusion protein using protein A beads [20], [28]; 2) increase the immunogenicity of the Fc-containing protein [15], possibly through the binding of Fc in the protein to the Fc receptor (FcR) on the antigen-presenting cells (APCs) [29]; and 3) increase the *in vivo* half-life and stability of the Fc-containing protein by the formation of stable dimeric or tetrameric conformational structure [30].

As expected, the purified S377-588-Fc fusion protein could form a stable dimeric or tetrameric structure being able to bind efficiently to sDPP4, as well as cell-surface receptor DPP4 (Fig. 2), resulting in the potent inhibition of MERS-CoV infection in DPP4-expressing cells (Fig. 3). These results suggest that the formation of dimer or tetramer by S377-588-Fc does not hinder the binding to its receptor DPP4. Thus, S377-588-Fc appeared much more potent than S358-588-Fc and S367-588 in inhibiting MERS-CoV infection [16], [19], indicating that removal of the S358-376 fragment might have an enhancing effect on RBD-mediated anti-MERS-CoV activity.

Like antibodies, recombinant proteins can also be used as clinical therapeutics for the treatment of cancer [31]–[33]. The peptide-based HIV entry inhibitor enfuvirtide has been used for treating HIV infection [34]–[36]. Because of its potent inhibitory activity on MERS-CoV entry, the recombinant S377-588-Fc protein may be further developed as a MERS-CoV entry inhibitor for treatment of MERS-CoV infection in the acute stage. The major concern for use of protein drugs in clinics is their production of antibodies, which may suppress drug efficacy. However, this may not be a concern for the MERS-CoV S-RBD protein-based therapeutic. First, the human IgG Fc in the S377-588-Fc fusion protein is not expected to induce antibodies in humans. Second, S377-588-Fc will be mainly used for urgent life-saving treatment at the early stage of MERS-CoV infection. Even if it does elicit anti-RBD antibodies in the patient several weeks after use, the patient may have passed through the most dangerous acute period. The antibodies induced by the protein drug may be actually helpful for the patient to neutralize the re-entered MERS-CoV.

**Figure 6:**
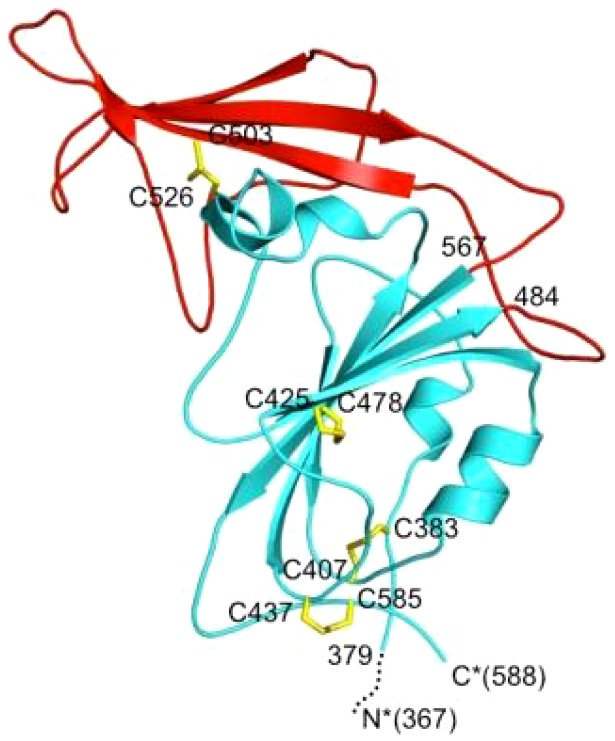
Crystal structure of MERS-CoV RBD (PDB 4L3N) [19]. The core structure of RBD is in cyan, and RBM in red. Helices are drawn as cylinders, and strands as arrows. Disulfide bond linked cysteines are shown as sticks in yellow. The N- and C-termini of the crystallized protein fragment are shown as N* and C*, respectively. The disordered region on the N-terminus of the RBD is shown as a dashed line. The boundaries of the RBD and of the RBM are labeled by their residue numbers.

We then tested immunogenicity of the truncated RBD of MERS-CoV S protein in inducing anti-MERS-CoV immune responses and neutralizing antibody responses in vaccinated mice. Notably, S377-588-Fc elicited elevated MERS-CoV RBD-specific IgG antibody responses after each boost, reaching the highest level after the 3^rd^ vaccination (Fig. 4). These mouse antisera exhibited potent neutralizing antibody responses with neutralizing antibody titers of ∼1:1,500, which are much higher than those induced by S377-662-Fc [15]. These data suggest that the truncated RBD fragment containing residues 377-588 is more immunogenic in inducing RBD-specific neutralizing antibody response than the longer RBD fragment containing residues 377-662, possibly because the C-terminal region spanning residues 589-662 contains non-neutralizing epitopes, which may compete with the neutralizing epitopes in the residues 377-588 region in eliciting antibody responses. In addition, the residues 589-662 fragment may destabilize the core conformation of the RBD. Analysis of the crystal structure of the RBD suggests that cysteines 379 and 585 are both involved in stabilizing the tertiary structure of the RBD by forming disulfide bonds with their respective cysteine partners. A few extra residues (e.g., residues 377-378 at the N-terminus and residues 586-588 at the C-terminus) may be needed to flank these cysteines for the proper formation of the disulfide bonds (Fig. 6) [19]. However, the extended region at the C-terminus (residues 589-662) may have adverse effect on tertiary conformation of the core structure (residues 379-585). Therefore, this truncated RBD fragment (residues 377-588) may have reached the optimal length of RBD that contains the intact functional domain (RBM: residues 484-567) and major neutralizing epitopes, and maintains a stable core conformation.

Our previous studies have shown that SARS-CoV S-RBD-induced antibodies are effective in inhibiting SARS-CoV S-RBD-binding to ACE2, the receptor of SARS-CoV [24]. Similarly, we demonstrated here that antisera of mice vaccinated with the truncated S-RBD (S377-588) of MERS-CoV could also efficiently block the binding of MERS-CoV S-RBD to its receptor, DPP4, indicating that antibodies in the mouse antisera can neutralize MERS-CoV infection, possibly through inhibition of virus-receptor binding. Therefore, both S-RBD protein and anti-RBD antibodies can be developed as therapeutics to treat MERS-CoV infection.

It is necessary to further test the therapeutic and preventive effects of this truncated S-RBD in an animal model. Thus far, however, no small animal model has been established for a MERS-CoV challenge study [37]. While the lung cells of Syrian hamster express the receptor of MERS-CoV, it was reported that this animal model was not susceptible to the induction of MERS-CoV infection [38]. Rhesus macaques could generate pneumonia-like symptoms within 24 hours of MERS-CoV infection [39]; however, the macaque system is not as widely applicable as a small animal model. Therefore, we are awaiting the establishment of a small animal model in order to carry out viral challenge studies.

In conclusion, we have identified a 212-amino acid truncated RBD in the S1 subunit of MERS-CoV S protein fused with human IgG Fc. This protein, denoted S377-588-Fc, can effectively bind to the viral receptor, DPP4, and potently inhibit MERS-CoV infection in DPP4-expressing cells. It can also induce strong RBD-specific antibody responses with potent neutralizing activity against infection from MERS-CoV. Therefore, this truncated S-RBD linked with Fc shows potential for further development as both therapeutic and vaccine for the treatment and prevention of MERS-CoV infection.

## Materials and Methods

### Ethics statement

Six- to eight-week-old female BALB/c mice were used in the study. The animal studies were carried out in strict accordance with the recommendations in the Guide for the Care and Use of Laboratory Animals of the National Institutes of Health. The protocol was approved by the Committee on the Ethics of Animal Experiments of the New York Blood Center (Permit Number: 194.14).

### Construction, expression and purification of recombinant proteins

The construction, expression and purification of recombinant MERS-CoV S proteins were done as previously described with some modifications [40]. Briefly, genes encoding residues 377-588 of MERS-CoV S protein were amplified by PCR using codon-optimized MERS-CoV S sequences (GenBank: AFS88936.1) as the template and then inserted into the pFUSE-hIgG1-Fc2 expression vector (hereinafter named Fc; InvivoGen, San Diego, CA). The MERS-CoV S1 (residues 18-725) plus 6× Histidine (His) was amplified as above and inserted into the pJW4303 expression vector (Jiangsu Taizhou Haiyuan Protein Biotech, Co., Ltd, China). The sequence-confirmed recombinant plasmids were transfected into 293T cells (ATCC, Manassas, VA) seeded 24 h before transfection, followed by replacement of culture medium by serum-free DMEM (Invitrogen, Carlsbad, CA) 8 h later and then collection of supernatant containing expressed protein 72 h post-transfection. The recombinant proteins were purified by Protein A affinity chromatography (GE Healthcare, Piscataway, NJ) (for proteins with Fc) or Ni-NTA Superflow (Qiagen, Valencia, CA) (for proteins with His tag), according to the manufacturers’ instructions.

The human DPP4 ectodomain (residues 39-766) was expressed and purified as previously described for human ACE2 ectodomain [11]. Briefly, human DPP4 ectodomain containing an N-terminal honeybee melittin signal peptide and a C-terminal 6× His tag was expressed in insect cells using the Bac-to-Bac expression system (Invitrogen), secreted into cell culture medium, and subsequently purified by Ni-NTA affinity column and Superdex200 gel filtration column (GE Healthcare).

### SDS-PAGE, Cross-linker and Western blot

The purified S377-588-Fc protein was analyzed by SDS-PAGE and Western blot as previously described [40]. Briefly, the proteins were either boiled for 10 min, or not boiled, and then separated by 10% Tris-Glycine SDS-PAGE gels, which were then stained with Coomassie Blue or transferred to nitrocellulose membranes for Western blot analysis. After blocking with 5% non-fat milk in PBST overnight at 4°C, the blots were incubated for 1 h at room temperature with MERS-CoV S protein-specific polyclonal antibodies (1:1,000) developed in our laboratories. After three washes, the blots were then incubated with horseradish peroxidase (HRP)-conjugated goat anti-mouse IgG (1∶5,000, Invitrogen) for 1 h at room temperature. Signals were visualized with ECL Western blot substrate reagents and Amersham Hyperfilm (GE Healthcare).

Protein cross-linker was done as previously described [40]. Briefly, 4.5 µg of purified S377-588-Fc protein was mixed with 20 µl of 0.1% glutaraldehyde (final concentration 4 mM) and left at room temperature in the dark for 3 h before SDS-PAGE using 6% Tris-Glycine gel and Western blot detection as described above.

### Co-immunoprecipitation assay

The binding between MERS-CoV S377-588-Fc and DPP4 was performed by co-immunoprecipitation assay as previously described [15]. Briefly, sDPP4 (10 µg) or DPP4-expressing Huh-7 cell lysates (5×10^7^/ml) were respectively incubated with MERS-CoV S377-588-Fc protein (10 µg) plus Protein A Sepharose Beads at 4°C for 1 h. After washing with lysis buffer and PBS, beads were boiled for 10 min and subjected to SDS-PAGE and Western blot as above for detecting DPP4 using an anti-DPP4 mAb (1 µg/ml, R&D Systems, Minneapolis, MN) or MERS-CoV S protein using MERS-CoV S1-specific polyclonal antibodies (1:1,000) developed in our laboratory.

### Flow cytometry

The binding of MERS-CoV S377-588-Fc to DPP4-expressing Huh-7 cells was also measured by flow cytometry. Briefly, cells (5×10^5^) were incubated with S377-588-Fc at various concentrations for 30 min at room temperature, followed by incubation with DyLight-488-labeled goat anti-human IgG antibody for 30 min and analysis by flow cytometry. Human IgG Fc at the same concentration was used as the control. The inhibition of S377-588-Fc binding to Huh-7 cells by mouse antisera was performed by co-incubating sera with MERS-CoV S377-588-Fc (0.5 µg/ml) and cells for 30 min at room temperature. After staining with DyLight-488-conjugated goat anti-human IgG, the mixture was analyzed by flow cytometry as above.

### Protein-mediated inhibition of MERS-CoV replication

Calu-3 cells that are highly susceptible to MERS-CoV [41] were incubated with serially diluted recombinant MERS-CoV S377-588-Fc or hIgG-Fc control protein for 1 h at 37°C. After removing media containing the protein, the cells were infected with MERS-CoV/Erasmus Medical Center (EMC)-2012 at a multiplicity of infection (MOI) of 0.1 for 1 h at 37°C. After thoroughly washing with PBS to remove unbound viruses, the infected cultures were replenished with MEM/2% FCS medium containing the aforementioned proteins diluted accordingly, followed by incubation for an additional 24 h before assessing the formation of CPE.

### Mouse vaccination and sample collection

This was done as previously described immunization protocols with some modifications [40]. Briefly, mice were prime-vaccinated (s.c.) with 10 µg/mouse of recombinant S377-588-Fc protein formulated with Montanide ISA 51 adjuvant (SEPPIC, Fairfield, NJ) and boosted twice with the same immunogen and adjuvant at 3-week intervals. Sera from pre-immunization and 10 days post-each vaccination were heat-inactivated at 56°C for 30 minutes and detected for S-specific antibody responses, neutralizing antibodies, or blockage of receptor binding.

### ELISA

ELISA was carried out to detect MERS-CoV S377-588-Fc protein binding to its receptor sDPP4 or MERS-CoV S-specific antibody responses in collected mouse sera as previously described [15]. Briefly, 96-well ELISA plates were respectively pre-coated with recombinant sDPP4 (2 µg/ml, for binding), MERS-CoV S377-588-Fc or S1-His protein (1 µg/ml, for antibody response) overnight at 4°C and blocked with 2% non-fat milk for 2 h at 37°C. Serially diluted MERS-CoV S377-588-Fc protein (for binding) or mouse sera (for antibody detection) were added to the plates and incubated at 37°C for 1 h, followed by four washes. Bound antibodies were respectively incubated with HRP-conjugated anti-human IgG (1:3,000, for binding, Invitrogen), or anti-mouse IgG, IgG1 and IgG2a (1:2,000, for antibody detection, Invitrogen), for 1 h at 37°C. The reaction was visualized by substrate 3,3′,5,5′-tetramethylbenzidine (TMB) (Invitrogen) and stopped by 1N H_2_SO_4_. The absorbance at 450 nm (A450) was measured by ELISA plate reader (Tecan, San Jose, CA).

### Neutralization assay

The standard micro-neutralization assay was used to quantify the neutralizing potential of each specimen, as previously described [15]. Briefly, serum samples were diluted at serial 2-fold in 96-well tissue culture plates and incubated at room temperature for an hour with ∼100 infectious MERS-CoV/EMC-2012 in each well before transferring the resultant mixtures to duplicate wells of confluent Vero E6 cells grown in 96-well tissue culture plates. After 72 h of incubation, when the virus control wells exhibited advanced virus-induced CPE, the neutralizing capacity of individual serum samples was assessed by determining the presence or absence of CPE. Neutralizing antibody titers were expressed as the reciprocal of the highest dilution of serum that completely (100%) inhibited virus-induced CPE in at least 50% of the wells.
