# Correction: A Truncated Receptor-Binding Domain of MERS-CoV Spike Protein Potently Inhibits MERS-CoV Infection and Induces Strong Neutralizing Antibody Responses: Implication for Developing Therapeutics and Vaccines

**DOI:** 10.1371/journal.pone.0278474

**Published:** 2022-12-05

**Authors:** Lanying Du, Zhihua Kou, Cuiqing Ma, Xinrong Tao, Lili Wang, Guangyu Zhao, Yaoqing Chen, Fei Yu, Chien-Te K. Tseng, Yusen Zhou, Shibo Jiang

During figure preparation, the incorrect image was inadvertently used to prepare the Fig 3 hlgG-Fc CPE 4 1.6μm/ml panel. The updated Fig 3 below has been amended so that the correct hlgG-Fc CPE 4 1.6μm/ml panel is presented.

**Fig 3:**
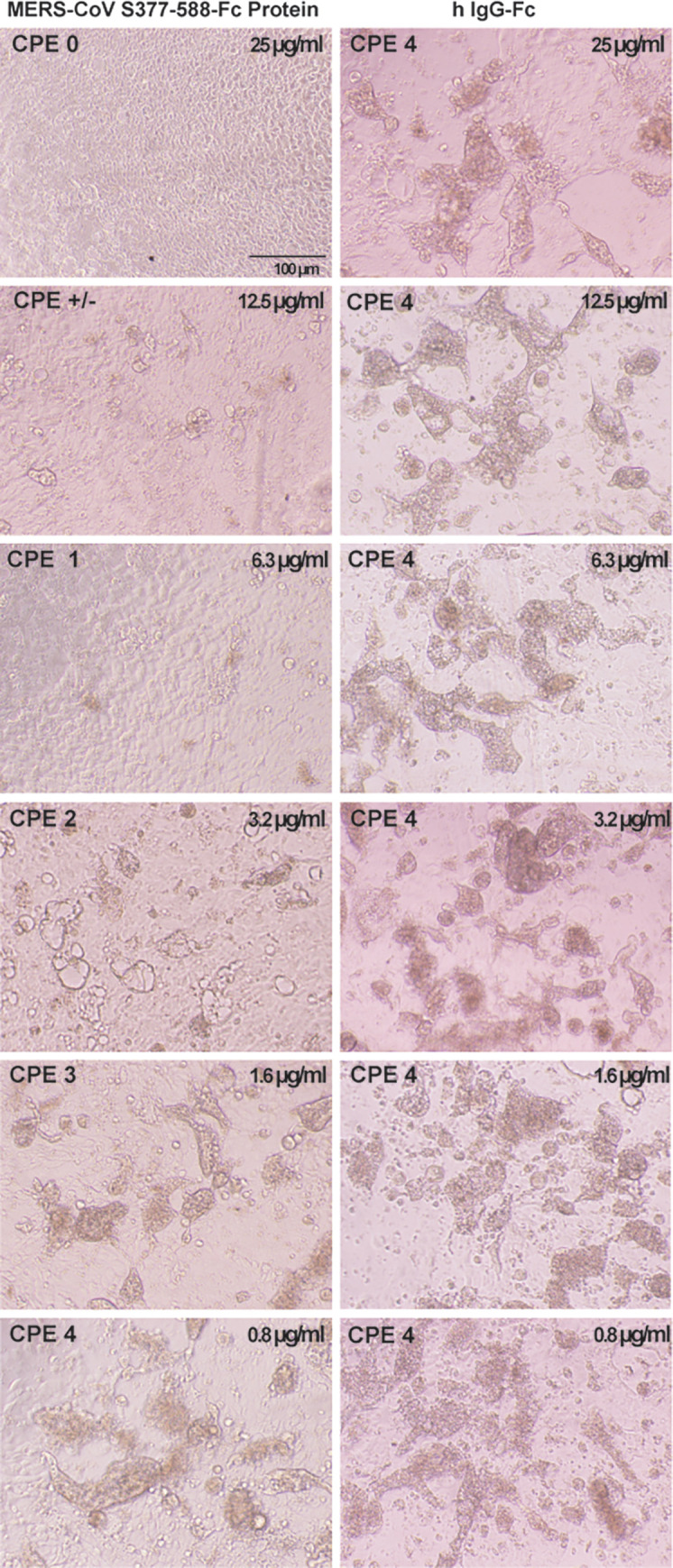


The original data underlying the results presented in this article remain available upon request from the corresponding author.

In addition, corresponding author SJ informed the journal that co-corresponding author YZ is deceased. Any future correspondence related to this article should be directed to SJ, and their updated contact details are shibojiang@fudan.edu.cn.

## References

[1] Du L, Kou Z, Ma C, Tao X, Wang L, Zhao G, et al. (2013) A Truncated Receptor-Binding Domain of MERS-CoV Spike Protein Potently Inhibits MERS-CoV Infection and Induces Strong Neutralizing Antibody Responses: Implication for Developing Therapeutics and Vaccines. PLoS ONE 8(12): e81587. 10.1371/journal.pone.0081587 24324708 PMC3852489

